# In-hospital mortality among patients with congenital heart disease undergoing noncardiac procedures: machine learning analysis of a multicentre study

**DOI:** 10.1016/j.bjao.2026.100581

**Published:** 2026-06-30

**Authors:** Michael T. Kuntz, Mikaela J. Mari, Steven J. Staffa, Vannessa Chin, Nina Deutsch, David Faraoni, Wanda C. Miller-Hance, Susan C. Nicolson, Martina Richtsfeld, Viviane G. Nasr

**Affiliations:** 1 Division of Pediatric Cardiac Anesthesia, Department of Anesthesiology, Monroe Carell Jr. Children’s Hospital at Vanderbilt, Nashville, TN, USA; 2 Department of Anesthesiology, Critical Care, and Pain Medicine, Boston Children’s Hospital, Harvard Medical School, Boston, MA, USA; 3 The Hospital for Sick Children, Department of Anesthesiology and Pain Medicine, Toronto, ON, Canada; 4 Department of Anesthesiology, Pain and Perioperative Medicine, Children’s National Hospital, The George Washington University School of Medicine and Health Sciences, Washington, DC, USA; 5 Department of Anesthesiology, Perioperative and Pain Medicine, Texas Children’s Hospital, Baylor College of Medicine, Houston, TX, USA; 6 Division of Cardiothoracic Anesthesia, Department of Anesthesiology and Critical Care Medicine, The Children’s Hospital of Philadelphia, Perelman School of Medicine at the University of Pennsylvania, Philadelphia, PA, USA; 7 Division of Pediatric Cardiac Anesthesia, Department of Anesthesiology, University of Minnesota Masonic Children’s Hospital, Minneapolis, MN, USA

**Keywords:** congenital heart disease, mortality, noncardiac surgery, paediatric, risk factors

## Abstract

**Background:**

Improved survival for children with congenital heart disease (CHD) has resulted in an increasing number presenting for noncardiac procedures. Several factors are associated with mortality in these patients, including severity of heart disease, preoperative inotropic support, preoperative ventilatory support, emergency procedure, and comorbidities. Prior literature has relied on multivariable regression analyses. The aim of this analysis was to utilise machine learning (ML) methodology to determine risk factors for in-hospital mortality after noncardiac procedures in paediatric patients with CHD.

**Methods:**

Seven paediatric centres collected data between January 2021 and December 2022 involving patients <21 yr of age with CHD undergoing noncardiac procedures. Both the random forest and eXtreme Gradient boosting machine models were tested, with the optimal model chosen based on the area under the precision recall curve in the validation set.

**Results:**

Of 5977 patients, 1.74% (*n=*104) had in-hospital mortality; among 9837 surgical encounters, 1.48% (*n=*146) were associated with in-hospital mortality. The top features contributing to mortality prediction in the eXtreme Gradient boost model were similar to defined factors in previous multivariable regression analyses: pre-procedural inotropic support; duration of anaesthesia; severe cardiac disease; prematurity; white race; emergent procedure; gender; inpatient procedure; black race; weekend procedure; and gastrointestinal, neurological, haematological, and respiratory comorbidities. A decision curve analysis shows benefit to understanding patients at increased risk of mortality when the probability of mortality is between 0% and 60%.

**Conclusions:**

The risk factors shown in our dataset using ML methods are similar to those demonstrated in our prior work utilising logistic regression. Ongoing awareness of these mortality predictors is essential to appropriate care planning.

**Editor’s key points**

• Children with congenital heart disease remain at increased risk of perioperative complications and mortality during noncardiac procedures.
• This machine learning analysis builds on the authors prior regression-based analyses to characterise in-hospital mortality and associated risk factors in children with congenital heart disease undergoing noncardiac procedures.
• Mortality was comparable to rates previously reported for this cohort.
• Risk factors identified by machine learning were consistent with those found in prior logistic regression analyses.
• Recognition of mortality predictors remains essential to perioperative care planning. Future work should identify modifiable risk factors to improve outcomes.

The incidence of any form of congenital heart disease (CHD) is estimated at 75 per 1000 live births, with severe disease occurring in 2.5–3 per 1000 live births.^1^ Because of advancements in medical care and surgical and catheter-based interventions, an increasing number of children with CHD survive into adulthood.^2^ This has resulted in a growing subset of this population requiring noncardiac procedures.^3^

Despite such advances, multiple studies have shown that CHD remains strongly associated with an increased risk of perioperative complications and mortality during admissions for noncardiac procedures.^4^^,^^5^ Children with CHD have been well-established to have a higher rate of mortality after noncardiac surgery compared to children without CHD.^6^ Multiple studies have examined factors associated with mortality in this population during hospitalisations for noncardiac surgical procedures, demonstrating extracardiac birth defects, severity of heart disease, ventricular dysfunction, preoperative cardiopulmonary resuscitation, need for inotropic support, mechanical ventilation, emergency procedure, comorbidity of other organ systems, and prolonged length of stay to be associated.^7^, ^8^, ^9^, ^10^ Patients with hypoplastic left heart syndrome, pulmonary hypertension, left ventricular outflow obstruction, and cardiomyopathy have also been shown to be at higher risk of perioperative complications including cardiac arrest and mortality compared with other lesions.^11^, ^12^, ^13^ In addition, patients with CHD who sustain cardiac arrest in the peri-anaesthetic period are at particularly high risk of mortality.^12^

We previously evaluated the incidence of and risk factors for intraoperative cardiac events for paediatric patients with CHD undergoing noncardiac procedures using a multicentre registry.^14^ With an additional year of data collected, we sought to use the multicentre database to examine all-cause inpatient mortality for this patient population. Our prior analysis, like much of the related literature, is based on multivariable regression models. Machine learning (ML), however, has emerged as a powerful tool to analyse datasets involving potentially non-linear relationships.^15^^,^^16^ Our primary aim is to use a ML analysis, a methodology which has not been broadly used to study this patient population, to determine all-cause in-hospital mortality and describe associated factors for patients with CHD undergoing noncardiac procedures. Doing so will allow us to compare factors demonstrated in this analysis with those shown in historical multivariable regression models.

## Methods

Data was gathered with a multi-institutional registry involving 10 paediatric academic medical centres. The study was approved by the Institutional Review Board (IRB) of the corresponding author’s institution (coordinating centre) and registered at Clinical Trials.gov (NCT 04604418). All centres received IRB approval, with the option of either using a cooperative agreement with the coordinating centre’s IRB (primary investigator VGN) or having the protocol reviewed by their local IRB. Patients were prospectively identified, and data was collected retrospectively at each centre by review of the medical record and submitting through REDCap to the coordinating centre.^17^

### Outcomes

The primary aim of this analysis was to utilise ML methodology to determine factors that influence in-hospital mortality after noncardiac procedures in paediatric patients with CHD. Doing so will allow us to compare factors demonstrated in this analysis with those shown in previously published multivariable regression models.^9^^,^^18^

### Study population

Seven of 10 centres, all quaternary care academic medical centres, contributed at least one full year of data (Supplementary Table 1). Three centres were not included because of investigator relocation, lack of sufficient data contribution, or both. Eligible patients included all patients with a history of CHD (Supplementary Table 2; e.g. included structural heart disease, cardiomyopathy, pulmonary vein stenosis; excluded patent foramen ovale only, isolated left superior vena cava to coronary sinus, isolated right aortic arch) from birth to 21 yr of age undergoing noncardiac procedures (surgical or nonsurgical) between 1 January 2021 and 31 December 2022. Cardiac surgical procedures (including pacemaker related interventions), cardiac catheterisation (diagnostic or interventional), and electrophysiology studies were excluded. Patients with multiple procedures within the study period had separate entries for each procedure.

### Study variables

Patient characteristic information collected included ASA-Physical Status Classification (ASA-PS), as determined by the attending anaesthesiologist the day of the procedure, gender, history of premature birth (under 37 weeks gestational age), and race. The medical record was reviewed for recent respiratory illness, preoperative mechanical ventilator support, preoperative inotropic support, cardiopulmonary resuscitation in the last 7 days, and current use of oral cardiac medications including anti-pulmonary hypertensives. The medical history was reviewed for sleep apnoea, chromosomal abnormalities (trisomy 13, 18, 21; DiGeorge and Williams Syndrome) and other comorbid conditions (including respiratory, neurological, endocrine, haematological, hepatic, gastrointestinal, genitourinary, and neoplastic conditions). CHD severity was classified based on the American College of Surgeons National Surgical Quality Improvement Program (ACS-NSQIP) guidelines into minor, major, or severe disease.^19^

Procedural information collected included emergent nature of procedure, clinical setting (inpatient or outpatient), day of procedure (weekday or weekend), type of procedure (surgical or nonsurgical), and duration of anaesthesia. Anaesthetic information including type of induction (inhalation, i.v., i.m.), anaesthetic technique (general, neuraxial, regional, local, or monitored anaesthesia care), and intra-operative airway management were also collected. Anaesthesiologists were classified as paediatric cardiac anaesthesiologists by training or practice, noncardiac paediatric anaesthesiologists whose clinical practice is more than 80% caring for children, noncardiac paediatric anaesthesiologists whose clinical practice is 50%–80% caring for children, noncardiac paediatric anaesthesiologists whose clinical practice is less than 50% caring for children, adult cardiac anaesthesiologists, or anaesthesiologists without paediatric or cardiac specialisation.

### Statistical analysis

Data are summarised in the full cohort using medians and inter-quartile ranges for continuous data and frequencies and percentages for categorical and binary data. An exploratory data analysis (EDA) was performed to examine the distribution of all variables, variables with missing data, and to evaluate the mortality rate for each variable. Denominators are presented for variables with missing data in the summary tables. The EDA was performed to understand the empirical associations between each factor and mortality. These results help to understand the data before proceeding with ML modelling. We chose to exclude ASA-PS from the model as the majority of patients were ASA-PS 3 or higher. ML modelling was performed using Stata (version 19, StataCorp LLC, College Station, Texas) with ‘h2oml’, with a 70%/30% split of the data for model training and for model validation. The 70%/30% split was selected to retain a large sample size in the validation set to include a meaningful number of mortality events. Features were included in the ML modelling as categorical and binary features, without normalisation, with the exception of duration of anaesthesia which was included as a raw continuous variable. ML models examined during model development were random forest and eXtreme Gradient boosting machine (XG Boost). These are ML models for binary classification based on decision trees. Models were tuned with three-fold cross validation using a random grid search for hyperparameter tuning, based on optimising the area under the precision recall curve (AUC-PR). Hyperparameters included in the tuning were number of trees (ranging from 30 to 70 by five), tree depth (ranging from three to 10 by one), and learning rate (ranging from 0.05 to 0.3 by 0.05). Hyperparameters utilised at their default values included sampling rate and minimum observations for leaf split. These tree-based ML models automatically performed missing data imputation by minimising the loss function for every possible split in the branch of the decision tree. The comparison of ML model performance in the validation set was performed by examining the following metrics: area under the receiver operating characteristic curve (AUC-ROC), AUC-PR, mean square error (MSE), root mean square error (RMSE), log loss, mean class error, Gini coefficient, accuracy, precision (or positive predictive value), negative predictive value, recall (or sensitivity), specificity, and F1 score. The ML model produces predicted probabilities of mortality, and the optimal cutoff threshold for the confusion matrix was identified based on maximising the F1 score. The identification of the optimal ML model was based on maximising the AUC-PR in the validation set. AUC-PR was examined rather than AUC-ROC because of substantial class imbalance. The most important contributing risk factors or features for predicting mortality were determined based on the magnitude of information gain. An optimal cutoff for duration of anaesthesia in distinguishing between patients with and without mortality was determined using ROC analysis and identifying the cutoff that maximises the combination of sensitivity and specificity.

Our ML classifiers utilise tree-based methodology that can help to uncover non-linear predictive associations that may not be detected by traditional linear regression models. Furthermore, the ML models are not limited by events per variable criteria. As a sensitivity analysis, a multivariable mixed-effects logistic regression model was fit using the same variables included in the ML modelling. Mixed-effects modelling was used to account for clustering of patients within centres and multiple encounters per patient. The AUC-ROC was calculated for this traditional multivariable regression modelling in the validation set. Results of this regression analysis are presented as adjusted odds ratios, 95% confidence intervals (CIs), and *P*-values.

## Results

A total of 9837 unique surgical encounters were included in the final analysis. There were 146 (1.48%) surgical encounters with associated in-hospital mortality; among the 5977 unique patients in the registry, there were 104 in-hospital mortalities (1.74%). Patient characteristics and medical history, including ACS-NSQIP risk stratification and cardiac disease history, are presented in Table 1. Most patients had minor cardiac disease (61.4%), and the highest mortality rate was observed for encounters for patients with severe cardiac disease (7.65%) compared with major (1.42%) or minor (0.53%) cardiac disease. Procedural characteristics are shown in Table 2 and anaesthetic management in Table 3. Encounters associated with emergent procedures had a mortality rate of 4.43%. Encounters associated with inpatient procedures had a higher mortality rate (4.05%) than outpatient encounters (0.08%). Weekend procedures had higher associated mortality (6.08%) than weekday procedures (1.30%).

**Table 1:** Patient characteristics and medical history. Data are presented as *n* (%). Denominators are indicated for the amount of non-missing data. ACS-NSQIP, American College of Surgeons, National Surgical Quality Improvement Program; ASA-PS, ASA Physical Status Classification; CPR, cardiopulmonary resuscitation.

Variable	Total	Mortality rate *n* (row %)
	(*N*=9837)	
**All patients**	**9837**	**146 (1.48%)**
**ASA-PS**		
1	55/9783 (0.6%)	0 (0%)
2	1552/9783 (15.9%)	1 (0.06%)
3	6022/9783 (61.6%)	20 (0.33%)
4	2094/9783 (21.4%)	109 (5.21%)
5	60/9783 (0.6%)	15 (25%)
**History of prematurity (under 37 weeks)**	3295/9703 (34%)	60 (1.82%)
**Gender**		
Male	5347/9787 (54.6%)	76 (1.42%)
Female	4440/9787 (45.4%)	69 (1.55%)
**Race**		
White	5554 (56.5%)	55 (0.99%)
Black or African American	1083 (11%)	31 (2.86%)
Other	1325 (13.5%)	13 (0.98%)
**Medical history**		
**Sleep apnoea**	1295/9444 (13.7%)	16 (1.24%)
**Chromosomal abnormalities**	4257/9765 (43.6%)	56 (1.32%)
**Chronic medical conditions**		
Respiratory	5410 (55%)	133 (2.46%)
Neurological	4216 (42.9%)	57 (1.35%)
Endocrine	2298 (23.4%)	43 (1.87%)
Haematological	2821 (28.7%)	70 (2.48%)
Hepatic	780 (7.93%)	29 (3.72%)
Gastrointestinal	6043 (61.4%)	110 (1.82%)
Genitourinary	2134 (21.7%)	47 (2.20%)
Neoplasm	464 (4.9%)	3 (0.65%)
**Preoperative ventilation support**	1172 (11.9%)	81 (6.91%)
**Preoperative inotropic support**	535/9757 (5.5%)	78 (14.58%)
**Concurrent respiratory illness**	957/9683 (9.9%)	21 (2.19%)
**ACS-NSQIP risk stratification**		
Minor	5088/8285 (61.4%)	27 (0.53%)
Major	2047/8285 (24.7%)	29 (1.42%)
Severe	1150/8285 (13.9%)	88 (7.65%)
**CPR within last 7 days**	79/9729 (0.8%)	9 (11.39%)
**Current oral cardiac medications**	3265/9767 (33.4%)	99 (3.03%)
**Anti-pulmonary hypertensives (number)**		
0–1	9726 (98.9%)	141 (1.45%)
2–3	111 (1.1%)	5 (4.50%)

**Table 2:** Procedure characteristics. Data are presented as *n* (%) or median (inter-quartile range). Denominators are indicated for the amount of non-missing data.

Variable	Total	Mortality rate *n* (row %)
	(*N*=9837)	
**Emergent**	971/9573 (10.1%)	43 (4.43%)
**Patient type**		
Inpatient	3459/9781 (35.4%)	140 (4.05%)
Outpatient	6322/9781 (64.6%)	5 (0.08%)
**Procedure scheduled**		
Weekday	9399/9777 (96.1%)	122 (1.30%)
Weekend	378/9777 (3.9%)	23 (6.08%)
**Procedure type**		
Surgical	6104/9772 (62.5%)	70 (1.15%)
Nonsurgical	3668/9772 (37.5%)	75 (2.04%)
**Duration of anaesthesia (min)**	94 (62, 149)	106 (79, 160) among those who died 93 (62, 148) among those who survived
≤100 min	5335/9777 (54.6%)	63 (1.18%)
>100 min	4442/9777 (45.4%)	81 (1.82%)

**Table 3:** Anaesthetic management. Data are presented as *n* (%). Denominators are indicated for the amount of non-missing data. BiPAP, bilevel positive airway pressure; MAC, monitored anaesthesia care; NIPPV, non-invasive positive pressure ventilation; SGAW, supraglottic airway.

Variable	Total	Mortality rate *n* (row %)
	(*N*=9837)	
**Induction**		
Inhalation	5411/9728 (55.6%)	22 (0.41%)
Intravenous	4292/9728 (44.1%)	121 (2.82%)
Intramuscular	25/9728 (0.3%)	0 (0%)
**Anaesthetic management**		
General anaesthesia	9358 (95.1%)	130 (1.39%)
All others (neuraxial, regional, local, MAC, other)	479 (4.9%)	16 (3.34%)
**Neuromuscular blocking agents**	5465 (55.6%)	48 (0.88%)
**Airway management**		
Spontaneous natural airway and face mask	1979/8544 (20.7%)	23 (1.16%)
Tracheal tube or tracheostomy	6049/9544 (63.4%)	115 (1.90%)
Non-invasive (NIPPV/CPAP/BIPAP)	86/9544 (0.9%)	2 (2.33%)
Supraglottic airway (SGAW)	1356/9544 (14.2%)	3 (0.22%)
Other	74/9544 (0.8%)	1 (1.35%)
**Ventilation**		
Mechanical	4881/9456 (51.6%)	104 (2.13%)
Spontaneous and pressure support	4575/9456 (48.4%)	32 (0.70%)
**Anaesthesia staffing**		
**Practice of the anaesthesiologist**		
Paediatric anaesthesiologist covering paediatrics >80% of clinical time	7333/9771 (75.1%)	55 (0.75%)
Paediatric cardiac anaesthesiologist either by training or practice	2344/9771 (24%)	89 (3.80%)
Paediatric anaesthesiologist covering paediatrics 50%–80% of clinical time	51/9771 (0.5%)	1 (1.96%)
Other (adult cardiac, general, paediatric with <50% paediatric coverage by clinical time)	43/9771 (0.4%)	0 (0%)

**Fig 1:**
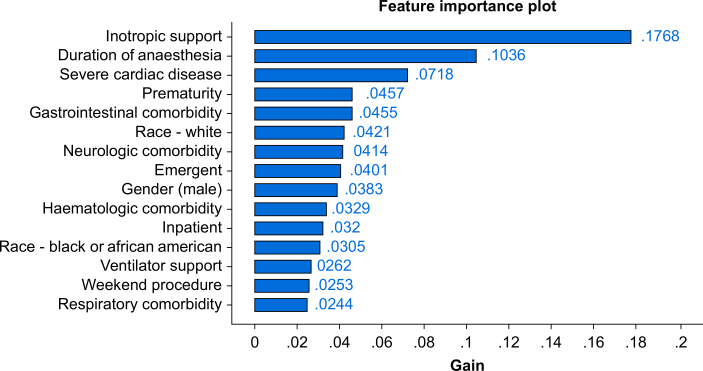
Feature importance plot for the XG Boost Model. The gain values show the relative contribution of each variable to predicting mortality.

**Fig 2:**
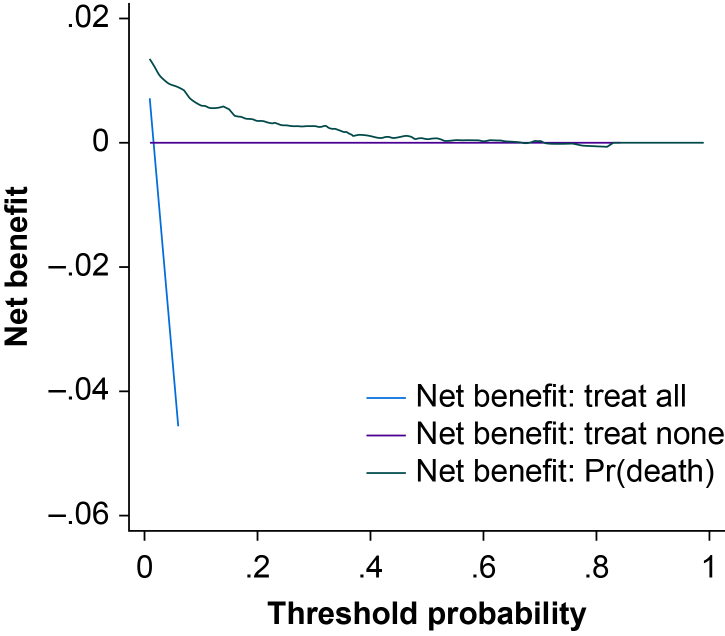
Decision curve analysis.

ML models were developed on the 70% training set (*n=*6909), and model performance was calculated using the 30% validation set (*n=*2928). In the model training set the mortality rate was 103/6909 (1.49%), and in the validation set the mortality rate was 43/2928 (1.47%). Table 4 shows strong performance of ML models in predicting mortality in the validation set. The optimal ML model based on maximised AUC-PR is the XG Boost model (AUC-PR=0.257, AUC-ROC=0.938). This model had a precision (positive predictive value) of 0.327, meaning that if the model predicted the patient to die, then this was associated with a 32.7% risk of mortality. In the XG boost model, the top features contributing to mortality prediction were pre-procedural inotropic support, duration of anaesthesia, severe cardiac disease, prematurity, gastrointestinal comorbidities, white race, neurological comorbidity, emergent procedure, gender, haematological comorbidity, inpatient procedure, black race, weekend procedure, and respiratory comorbidity (Figure 1). The gain values for preoperative inotropic support (0.1768), duration of anaesthesia (0.1036), and presence of severe cardiac disease (0.0718) were substantially higher than the remaining factors, which all had gain values less than 0.05. Decision curve analysis demonstrated that the final model provides net benefit across most threshold probabilities (Figure 2). The model provides benefit to understanding patients at increased risk of mortality when the probability of mortality is between 0% and 60%, but the model does not provide value when the probability of mortality is greater than 60%.

**Table 4:** Comparison of the performance of machine learning models for mortality (in the 30% validation set). All variables in the Exploratory Data Analysis were included as features in the machine learning models. The confusion matrix values (TP, TN, FP, FN), were obtained from a confusion matrix probability threshold that maximised F1 score (machine learning metric used to evaluate performance of a classification model). Tuned models implemented three-fold cross validation, and a random grid search of hyperparameter tuning, based on optimising AUC-PR. AUC-PR, area under the precision–recall curve; AUC-ROC, area under the receiver operating characteristic curve; FN, false negative; FP, false positive; MSE, mean squared error; RMSE, root mean squared error; TN, true negative; TP, true positive.

Metric	Random Forest	XG Boost
**Log loss**	0.101	0.052
**Mean class error**	0.224	0.308
**AUC-ROC**	0.842	0.938
**AUC-PR**	0.249	0.257
**Gini coefficient**	0.864	0.875
**MSE**	0.012	0.013
**RMSE**	0.111	0.112

**N in the validation set**	2928	2928
**TP**	25	17
**TN**	2796	2850
**FP**	89	35
**FN**	18	26

**Accuracy**	0.963	0.979
**Precision (positive predictive value)**	0.219	0.327
**Negative predictive value**	0.994	0.991
**Recall (sensitivity)**	0.581	0.395
**Specificity**	0.969	0.988
**F1 Score**	0.318	0.358

A sensitivity analysis using traditional multivariable regression (Supplementary Table 3) revealed similar independent predictors as identified by the ML model, including preoperative inotropic support, severe cardiac disease, inpatient status, weekend procedure, and respiratory comorbidity. Some features were identified by the ML model but not multivariable regression, including duration of anaesthesia, prematurity, emergent procedure, and preoperative ventilator support. A lower mortality rate for patients cared for by paediatric anaesthesiologists (who care for paediatric patients >80% of their clinical time) was identified in the regression analysis but not the ML model. The AUC-ROC for the multivariable regression model in the validation set is 0.937 (95% CI 0.908–0.965), compared with 0.938 for the XG Boost ML model.

## Discussion

Using a multicentre registry, we describe the incidence of mortality and associated risk factors for patients with CHD undergoing noncardiac procedures, applying a ML model to demonstrate patient factors most predictive of in-hospital mortality. ML has emerged as a powerful tool for interpreting large (or small) datasets. In particular, it may interpret nonlinear relationships more effectively than traditional modelling, albeit with relatively reduced interpretability.^15^^,^^16^ ML is also considered useful to identify predictors of binary outcomes (e.g., mortality).^20^ Although others have used ML methodology to examine outcomes in congenital heart surgery,^16^^,^^21^^,^^22^ ML analysis of outcomes for patients with CHD undergoing noncardiac procedures has not been undertaken, with the existing literature being driven by traditional multivariable statistical methods.

The overall mortality rate we observed (1.48% encounters associated with mortality; 1.74% of patients with mortality) is similar to a recent single institution study^18^ reviewing more than 1000 patients (30-day mortality 1.6%) but higher than a previous study examining trends in mortality over time.^3^ In this latter study utilising the Pediatric Health Information System (PHIS) database, the in-hospital mortality rate varied over the study period (2015–2019) between 1.06% and 1.3%, with an overall significant trend towards improvement. Not only was the overall mortality rate higher in our present study, but the mortality rate for inpatient encounters was also higher: between 3.02% and 3.38% in the prior PHIS study, compared with 4.05% in our current study. In a more recent study utilising the ACS-NSQIP database, the 30-day mortality rate was not different between the time periods of 2012–2016 and 2017–2022 for patients with any severity of CHD, with an overall mortality rate from 2012 to 2022 similar to our study (1.6%).^23^ We did, however, find a substantially higher mortality among encounters specifically with severe CHD: 7.65% compared with 3.38% in the ACS-NSQIP study. Major and minor CHD demonstrated lower mortality in our study, comparatively (1.42% vs 2.28% and 0.53% vs 0.83%, respectively). Notably, 13.9% of our cohort had severe CHD. By comparison, the ACS-NSQIP cohort included 5.4% of patients with severe CHD. The higher rate of severe CHD in our study is presumably a result of our participating centres being academic quaternary medical centres, compared with ACS-NSQIP which contains a wider variety of institution types. Notably, ACS-NSQIP examines a limited subset of noncardiac procedures, compared with our study which captured a wider variety of procedures. Finally, because ACS-NSQIP captures 30-day mortality, any events after this time would not be captured. Comparatively, we examined in-hospital mortality regardless of hospitalisation duration.

Patient factors contributing to mortality prediction have been explored previously. A risk stratification model aimed to predict postoperative mortality among patients with CHD undergoing noncardiac surgery showed the factors most predictive of postoperative mortality based on multivariable logistic regression included need for preoperative mechanical ventilation, acute or chronic kidney injury, preoperative cardiopulmonary resuscitation, preoperative inotropic support, surgery within the past 30 days, single-ventricle physiology, severe CHD, and emergency procedures.^9^ A large single institution study which also utilised multivariable logistic regression showed emergency procedure, preoperative renal abnormality, and preoperative mechanical ventilation to be associated with severe perioperative complications (cardiac arrest and mortality).^18^ Unsurprisingly, our ML analysis showed similar features as predictive of mortality, including preoperative inotropic support, preoperative mechanical ventilation, severe CHD, emergent procedure, and comorbidity of certain organ systems (gastrointestinal, neurological, haematological, and respiratory). Although it might seem obvious that patients with these factors are at high risk, appreciating the extent these factors contribute to mortality prediction is critical to allowing care teams to appropriately plan for perioperative care. Failure to evaluate and communicate patient level risk has even been described as an error trap in the care of the CHD population, underscoring the importance of having robust and reliable risk prediction tools.^24^ It is also worth noting that several of these factors, including preoperative inotropic support, preoperative mechanical ventilation, severe CHD, and emergent procedure were all shown to be associated with a higher rate of intraoperative cardiac events in prior analysis of our cohort, further emphasising the importance of recognising these factors during risk evaluation.^14^

Several other factors were also described by our model. Duration of anaesthesia was the feature with the second highest degree of importance when predicting postoperative mortality. Notably, we included duration of anaesthesia and not duration of procedure, as duration of procedure was related in a colinear fashion. In addition, anaesthesia time will better reflect patient intensity and may reflect overall acuity. Interestingly, the type of procedure was not highlighted as an important factor in the feature plot. This is consistent with a prior study showing that intrinsic surgical risk does not enhance mortality risk prediction for patients with CHD undergoing noncardiac surgery,^25^ even though it does enhance risk prediction for a non-CHD cohort.^26^

Certain races (namely, black and white) were found to be among the most important variables for predicting mortality. Prior targeted studies have also examined this relationship in detail, demonstrating worse outcomes after noncardiac surgery for black patients with minor and major CHD after propensity matching with white patients.^27^ Similarly, worse outcomes have been shown for non-white patients undergoing CHD surgery as well.^28^ Although feature importance plots do not indicate the direction of impact for each factor,^15^ such prior studies help contextualise this finding. Prematurity was another patient characteristic identified as important for mortality prediction in our present study. Although prematurity did not achieve significance for inclusion in a prior risk prediction model,^9^ others have shown an association between prematurity and mortality.^29^ In one single centre study, while prematurity was not predictive of death or cardiac arrest in multivariable analysis, it was associated with moderate perioperative complications (e.g., escalation of respiratory support, vasoactive medication use, readmission).^18^ The inclusion of prematurity in our model encourages its use as a risk stratification variable, despite the inconsistent inclusion in historical models.

Our dataset is unique in that it contains detailed anaesthesia staffing information, information often not contained in other large databases. Although the mortality rate for encounters involving paediatric cardiac anaesthesiologists was 3.80%, compared with 0.75% for paediatric anaesthesiologists who staff at least 80% paediatric patients, the provider type was not highlighted among the most important features for mortality prediction in ML analysis. This implies that patient factors, which inform anaesthesiologist assignment, may be the driving factors of mortality. Similarly, prior analysis of this registry showed a higher rate of intraoperative cardiac events for patients cared for by paediatric cardiac anaesthesiologists compared with paediatric anaesthesiologists in univariate analysis.^14^ However, after multivariable analysis, the adjusted odds ratio was no longer statistically significant. This supports that patient factors, not staff assignment, are the primary outcome drivers. Saettele and colleagues^30^ also presented a risk stratification algorithm that categorised patients with CHD into three tiers of risk before noncardiac procedures, allowing mindful preoperative planning including anaesthesiologist assignments. By doing so, they achieved similar outcomes between all levels of patient risk.^30^ This further emphasises the importance of strategic methods for assigning clinical coverage.

Our study utilised ML methodology to determine factors associated with mortality. In the decision curve for our model (Figure 2), it can be appreciated that net benefit to understanding mortality risk is achieved when the probability of mortality is between 0 and 60%. At mortality probabilities greater than 60% the model provides no net benefit. The model overall performed well, with the XG Boost achieving an accuracy of 97.9%, specificity of 98.8%, negative predictive value of 99.1%, and AUC-ROC 93.8%. In other words, if the model predicted no mortality, this was associated with a 99.1% chance of survival. The AUC-PR was selected as the metric for comparison of model performance because the AUC-ROC can be inflated due to class imbalance. The AUC-PR is modest in all ML models because of this class imbalance and low incidence of mortality. However, the probability of mortality given a ‘positive’ model-based prediction (the precision, or the positive predictive value) was 0.327 for the XG Boost model, indicating a 32.7% probability of mortality given a positive model prediction, which is substantially higher than the baseline 1.48% risk of mortality. Although this means two of three patients with predicted mortality will survive, relative overestimation of risk is more tolerable than under appreciation.

Although an advantage of the XG Boost is its strong predictive accuracy, a disadvantage of such ML models is lack of transparency that can be achieved through traditional statistical regression modelling, with effect sizes captured as adjusted odds ratios with CIs. The primary purpose of ML modelling is to calculate the most accurate predictors of the outcome, although the models are more complex and may be harder to interpret than traditional methods. The EDA helps to provide transparency of bivariate association between each feature and the outcome.

Our study has several limitations. First, as the data was collected by various study staff across multiple institutions, data definitions may have been variably interpreted despite regular meetings with site investigators to clarify standards and definitions. To mitigate this potential issue, the REDCap database was regularly audited, which allowed further opportunity to clarify definitions and recover missing data. Although the patients in this study were identified prospectively, much of the data collection occurred retrospectively and relied on the completeness and accuracy of clinical documentation. Although our study includes several institutions with variable patient volume, staffing arrangements, and clinical infrastructures, all participating centres were academic institutions. This may limit the overall generalisability of our findings to smaller private institutions. Validation of our model using an external data source would therefore be helpful and improve the generalisability of our findings. Although external validation would be preferred, availability of a data set with which to validate is limited, making external validation logistically impractical. Finally, the ML methodology is limited in that whereas non-linear associations may be detected more easily than with multivariable regression models, effect size and directionality are not transparent compared with odds ratios and CIs generated by multivariable techniques.

In conclusion, we have applied a ML methodology to a multicentre registry of patients with CHD having noncardiac procedures and described the factors that contribute to hospitalisation mortality prediction, most notably preoperative inotropic support, duration of anaesthesia, severe cardiac disease, prematurity, other organ system comorbid conditions (gastrointestinal, neurological, haematological, and respiratory), race, gender, emergent procedure, inpatient status, preoperative ventilator support, and weekend procedure. Although some of these factors have been identified in a prior multivariable model^9^ of hospitalisation mortality (preoperative inotropic support, severe cardiac disease, other organ comorbidities, emergent procedure, preoperative mechanical ventilation), the nature of our data set allowed us to identify other factors including duration of anaesthesia, prematurity, inpatient status, and weekend procedure. Ongoing awareness of these predictors will help clinicians appropriately plan perioperative care and disposition for this high-risk population.

## Author’s contributions

Conceptualisation: MK, SS, VC, ND, DF, WM, SN, MR, VN Data curation : MK, MM, VC, ND, DF, WM, SN, MR, VN Investigation: MK, VN Formal analysis: SS, VN Methodology: SS, VN Software: SS Drafting the manuscript: MK, MM, SS Reviewing and editing the manuscript: VC, ND, DF, WM, SN, MR, VN Supervision: VN

## Funding

Viviane Nasr received funding from the Institutional Centers for Clinical and Translational Research at Boston Children’s Hospital (Boston, USA).

## Declaration of interests

The authors declare that they have no conflict of interest.
