# Supplementary material for: In-hospital mortality among patients with congenital heart disease undergoing noncardiac procedures: machine learning analysis of a multicentre study
Source: BJA Open. 2026 Jun 30;19:100581. doi: 10.1016/j.bjao.2026.100581 (PMC13342874; doi:10.1016/j.bjao.2026.100581)
Supplement: Multimedia component 1 [file mmc1.docx]

| **Supplemental Table 1:** Participating Centers |
| --- |
| University of Minnesota Masonic Children's Hospital |
| Children's National Hospital, The George Washington University School of Medicine and Health Sciences |
| The Hospital for Sick Children |
| Monroe Carell Jr. Children’s Hospital at Vanderbilt |
| Boston Children’s Hospital |
| Texas Children's Hospital |
| The Children's Hospital of Philadelphia |

| **Supplemental Table 2:** Congenital Heart Disease Diagnoses for Inclusion and Exclusion | |
| --- | --- |
| **Include** | **Exclude** |
| - Structural heart disease, including any ventricular or atrial septal defect - Cardiomyopathy - Myocarditis only when structural disease present - Arrythmias, only when structural disease present - Pulmonary hypertension, only when structural disease present - Double orifice mitral valve - Unrepaired patent ductus arteriosus - Surgical or transcatheter patent ductus arteriosus closure - Cardiac mass or rhabdomyoma - Patient with genetic syndrome with cardiac sequelae (e.g., Williams or Marfan syndrome) - Congenital pulmonary vein stenosis - Vascular ring - Congenital complete heart block - Mid-aortic syndrome if structural heart disease also involved | - Chemotherapy related cardiac disease - Mediastinal mass - Patent foramen ovale only - Kawasaki disease - Isolated left superior vena cava to coronary sinus - Pulmonary hypertension without structural heart disease - Resolved pulmonary hypertension - Right aortic arch alone - Patent ductus arteriosus resolved spontaneously or after pharmacologic treatment - Mild ascending aortic dilation - Echocardiogram with mentioning atrial septal defect vs. patent foramen ovale - Interrupted inferior vena cava with azygous continuation with no other structural heart disease - Dyslipidemia - Aberrant subclavian artery - High output cardiac failure in the setting of Vein of Galen malformation - Mid-aortic syndrome without structural heart disease - Bicuspid aortic valve without stenosis or regurgitation |

| **Supplemental Table 3. Mixed-Effects Logistic Regression Analysis of Mortality** | |  |
| --- | --- | --- |
| **Variable** | **Adjusted Odds Ratio (95% CI)** | **P value** |
| **History of prematurity (under 37 weeks)** | 1.34 (0.52, 3.46) | 0.541 |
| **Gender - Female** | 1.26 (0.52, 3.02) | 0.606 |
| **Race** |  |  |
| White | 0.33 (0.12, 0.97) | **0.043*** |
| Black or African American | 0.96 (0.25, 3.65) | 0.958 |
| Other | 0.4 (0.09, 1.73) | 0.22 |
| **Sleep Apnoea** | 2.52 (0.56, 11.3) | 0.227 |
| **Chromosomal abnormalities** | 1.82 (0.72, 4.63) | 0.208 |
| **Chronic medical conditions** |  |  |
| Respiratory | 5.9 (1.57, 22.1) | **0.008*** |
| Neurologic | 0.8 (0.34, 1.89) | 0.616 |
| Endocrine | 0.51 (0.18, 1.51) | 0.227 |
| Hematologic | 1.71 (0.68, 4.29) | 0.254 |
| Hepatic | 2.26 (0.64, 7.93) | 0.204 |
| Gastrointestinal | 0.98 (0.39, 2.48) | 0.965 |
| Genitourinary | 0.95 (0.36, 2.55) | 0.925 |
| Neoplasm | 0.85 (0.06, 13) | 0.908 |
| **Preoperative ventilation support** | 2.02 (0.74, 5.51) | 0.168 |
| **Preoperative inotropic support** | 8.27 (3.15, 21.7) | **<0.001*** |
| **Concurrent respiratory illness** | 1.22 (0.37, 4.02) | 0.744 |
| **ACS-NSQIP Risk Stratification** |  |  |
| Minor | Reference |  |
| Major | 1.55 (0.44, 5.51) | 0.496 |
| Severe | 7.89 (2.22, 28.1) | **0.001*** |
| **CPR within last 7 days** | 4.86 (0.78, 30.2) | 0.09 |
| **Current Oral Cardiac Medications** | 1.43 (0.56, 3.62) | 0.456 |
| **Anti-Pulmonary Hypertensives (number)** |  |  |
| 0-1 | Reference |  |
| 2-3 | 1.27 (0.19, 8.62) | 0.806 |
| **Emergent** | 0.91 (0.33, 2.56) | 0.862 |
| **Patient Type** |  |  |
| Inpatient | 59.8 (8.47, 422.3) | **<0.001*** |
| Outpatient | Reference |  |
| **Procedure Scheduled** |  |  |
| Weekday | Reference |  |
| Weekend | 8.01 (1.86, 34.4) | **0.005*** |
| **Procedure Type** |  |  |
| Surgical | 1.22 (0.52, 2.83) | 0.651 |
| Non-surgical | Reference |  |
| **Duration of Anaesthesia (minutes)** | 0.99 per 30 minutes (0.98, 1.01) | 0.968 |
| **Induction** |  |  |
| Inhalation | Reference |  |
| Intravenous | 0.79 (0.29, 2.11) | 0.633 |
| Intramuscular | Cannot calculate |  |
| **Anaesthetic Management** |  |  |
| General anaesthesia | Reference |  |
| All others (neuraxial, regional, local, MAC, other) | 5.44 (0.77, 38.5) | 0.09 |
| **Neuromuscular Blocking Agents** | 1.08 (0.36, 3.29) | 0.888 |
| **Airway management** |  |  |
| Spontaneous natural airway and Face mask | Reference |  |
| Endotracheal tube or tracheostomy | 0.82 (0.16, 4.28) | 0.812 |
| Non-invasive (NIPPV/CPAP/BIPAP) | 2.45 (0.09, 68.6) | 0.597 |
| Supraglottic airway | 0.2 (0.01, 3.92) | 0.288 |
| Other | 9.66 (0.2, 456.9) | 0.249 |
| **Ventilation** |  |  |
| Mechanical | 2.85 (0.75, 10.8) | 0.125 |
| Spontaneous & Pressure Support | Reference |  |
| **Practice of the Anaesthesiologist** |  |  |
| Paediatric anaesthesiologist covering paediatrics >80% of clinical time | 0.36 (0.14, 0.94) | **0.036*** |
| Paediatric cardiac anaesthesiologist either by training or practice | Reference |  |
| Paediatric anaesthesiologist covering paediatrics 50-80% of clinical time | 2.42 (0.03, 189.2) | 0.692 |
| Other (adult cardiac, general, paediatric with < 50% paediatric coverage by clinical time) | Cannot calculate |  |
| Mixed-effects logistic regression was implemented with random effects to account for clustering of patients within centres and multiple encounters per patient. | | |
| *Statistically significant. |  |  |
| ACS-NSQIP, American College of Surgeons National Surgical Quality Improvement Program; CPR, cardiopulmonary resuscitation; MAC, monitored anaesthesia care; NIPPV, non-invasive positive pressure ventilation; CPAP, continuous positive airway pressure; BIPAP, bi-level positive airway pressure | | |
